# Label-Free Quantitative Proteomics Identifies Novel Plasma Biomarkers for Distinguishing Pulmonary Tuberculosis and Latent Infection

**DOI:** 10.3389/fmicb.2018.01267

**Published:** 2018-06-13

**Authors:** Huishan Sun, Liping Pan, Hongyan Jia, Zhiguo Zhang, Mengqiu Gao, Mailing Huang, Jinghui Wang, Qi Sun, Rongrong Wei, Boping Du, Aiying Xing, Zongde Zhang

**Affiliations:** ^1^Beijing Key Laboratory for Drug Resistant Tuberculosis Research, Beijing Tuberculosis and Thoracic Tumor Research Institute, Beijing Chest Hospital, Capital Medical University, Beijing, China; ^2^Changping Tuberculosis Prevent and Control Institute of Beijing, Beijing, China; ^3^Department of Tuberculosis, Beijing Tuberculosis and Thoracic Tumor Research Institute, Beijing Chest Hospital, Capital Medical University, Beijing, China; ^4^Department of Medical Oncology, Beijing Tuberculosis and Thoracic Tumor Research Institute, Beijing Chest Hospital, Capital Medical University, Beijing, China

**Keywords:** active tuberculosis, latent tuberculosis infection (LTBI), label-free quantitative proteomics, plasma protein, diagnostic model, ACT, AGP1, CDH1

## Abstract

The lack of effective differential diagnostic methods for active tuberculosis (TB) and latent infection (LTBI) is still an obstacle for TB control. Furthermore, the molecular mechanism behind the progression from LTBI to active TB has been not elucidated. Therefore, we performed label-free quantitative proteomics to identify plasma biomarkers for discriminating pulmonary TB (PTB) from LTBI. A total of 31 overlapping proteins with significant difference in expression level were identified in PTB patients (*n* = 15), compared with LTBI individuals (*n* = 15) and healthy controls (HCs, *n* = 15). Eight differentially expressed proteins were verified using western blot analysis, which was 100% consistent with the proteomics results. Statistically significant differences of six proteins were further validated in the PTB group compared with the LTBI and HC groups in the training set (*n* = 240), using ELISA. Classification and regression tree (CART) analysis was employed to determine the ideal protein combination for discriminating PTB from LTBI and HC. A diagnostic model consisting of alpha-1-antichymotrypsin (ACT), alpha-1-acid glycoprotein 1 (AGP1), and E-cadherin (CDH1) was established and presented a sensitivity of 81.2% (69/85) and a specificity of 95.2% (80/84) in discriminating PTB from LTBI, and a sensitivity of 81.2% (69/85) and a specificity of 90.1% (64/81) in discriminating PTB from HCs. Additional validation was performed by evaluating the diagnostic model in blind testing set (*n* = 113), which yielded a sensitivity of 75.0% (21/28) and specificity of 96.1% (25/26) in PTB vs. LTBI, 75.0% (21/28) and 92.3% (24/26) in PTB vs. HCs, and 75.0% (21/28) and 81.8% (27/33) in PTB vs. lung cancer (LC), respectively. This study obtained the plasma proteomic profiles of different *M.TB* infection statuses, which contribute to a better understanding of the pathogenesis involved in the transition from latent infection to TB activation and provide new potential diagnostic biomarkers for distinguishing PTB and LTBI.

## Introduction

Tuberculosis (TB) is a major global infectious disease causing high mortality and morbidity, with 1.7 million deaths and 10.4 million new cases worldwide in 2016. About 95% cases were PTB, which is mainly caused by *M.TB* transmission (WHO, 2017). Besides the active TB patients, about one-third of the world’s population is infected with *M.TB*, but remains asymptomatic, which is known as LTBI. It has been reported that 5–15% of LTBI individuals will eventually develop active TB (Getahun et al., 2015). Thus, the LTBI population may be a seedbed of TB in the community. Early diagnosis and prompt treatment of TB, not only for active TB but also for LTBI, are critical strategies for TB control. However, the current routine methods demonstrate some limitations in identifying TB, especially in discriminating active TB from LTBI. *M.TB* culture is the gold standard method for TB diagnosis but require 4–8 weeks for results (Brodie and Schluger, 2005). The microscopic examination of sputum allows recognition of the bacilli, but only if sputum contains at least 5 × 10^3^ bacilli/mL (Kumar et al., 2014). The interferon gamma release assays (IGRAs) are now a promising method to diagnose *M.TB* infection but cannot distinguish active TB from LTBI, especially in the suspected TB patients with clinical respiratory symptoms (Sester et al., 2011). Therefore, aforementioned methods do not satisfy the requirement to definitively diagnose patients with early-stage active pulmonary TB. Consequently, a panel of easily measured biomarkers with high diagnostic accuracy is of paramount importance for global TB control.

In recent years, great efforts have been made to address this problem. Since peripheral blood is easy to collect, screening and identifying biomarkers in plasma or serum is an effective method of disease diagnosis. Proteins are the ultimate players in biological activities. Pathogenic mechanisms between TB and host during *M.TB* invasion are based on protein expression and protein–protein or protein–nucleic acid interactions. These TB-associated proteins may be used as potential diagnostic markers for identifying active TB. High-throughput spectrometric techniques have advanced rapidly and provide a broad platform for TB research and biomarker discovery (Liu et al., 2013; Xu et al., 2014, 2015). The label-free proteomics technique could be a promising method to discover protein markers in cancers (Fan et al., 2015; Gamez-Pozo et al., 2015). However, few studies have used this technique to examine the plasma proteomic profile of PTB and LTBI individuals. Furthermore, most studies have failed to establish a simple visual model to distinguish active TB from LTBI, especially a model with high diagnostic performance in a blind testing set.

In this study, we applied a label-free quantitative proteomics technique to identify candidate plasma biomarkers associated with PTB. A new proteomic profile for distinguishing PTB from LTBI and HCs was generated, and an effective diagnostic model with a relatively high accuracy for distinguishing PTB from LTBI and HC was constructed and further validated. Furthermore, this diagnostic model showed a relatively good sensitivity and specificity in distinguishing PTB and LC. These results also provide a new database of proteins that can be used to understand the pathogenesis from latent infection to TB reactivation.

## Materials and Methods

### Study Population

This study was carried out in accordance with the recommendations of the Helsinki Declaration and its later amendments or comparable ethical standards, the Ethics Committee of Beijing Chest Hospital, Capital Medical University. The protocol was approved by the Ethics Committee of Beijing Chest Hospital, Capital Medical University. All subjects gave written informed consent in accordance with the Declaration of Helsinki.

The PTB patients were recruited from Beijing Chest Hospital between December 2012 and October 2014. All PTB patients exhibited typical TB clinical symptoms, TB lesion revealed by chest radiograph, at least two consecutive positive sputum smears, and/or a positive sputum culture. The PTB patients enrolled in the discovery set for proteomics analysis had not received anti-TB treatment. The majority of the PTB patients in the training set and blind test set had received anti-TB treatment for no more than 14 days; only 7 patients had received anti-TB treatment for 14–26 days. LTBI individuals and HC were recruited from a large TB screening campaign in Beijing between November 2012 and December 2014. LTBI individuals satisfied the following criteria: positive TST and T-SPOT.*TB* results, normal chest radiograph, and without any clinical evidence of active TB and other diseases. Since most of the participants in our study were BCG vaccinated, the TST/T-SPOT.*TB* two-step strategy was used in accordance with previous studies (Berry et al., 2010; Zak et al., 2016) because confirmatory T-SPOT.*TB* can highly reduce the false positive rate due to BCG vaccination or NTM infection in the initial TST. HCs were people with negative TST and T-SPOT.*TB* tests, normal chest radiograph and no clinical symptoms of diseases. Histologically or cytologically methodology proven LC patients were recruited from Beijing Chest Hospital between October 2013 and December 2014. Individuals with positive human immunodeficiency virus (HIV), positive hepatitis B virus (HBV) or hepatitis C virus (HCV), diabetes, or severe autoimmune diseases; those who took immunosuppressive or immunopotentiator agents; and those who were pregnant or lactating were excluded.

### Blood Sample Collection

Whole blood samples were collected in heparin-containing vacutainer tubes, and then centrifuged at 2,000 rpm for 10 min at 4°C to obtain the fresh plasma. The plasma was immediately aliquoted into sterile centrifuge tubes and frozen at -80°C for future research. No samples were frozen and thawed repeatedly.

### Sample Preparation for Mass Spectrometry

In the discovery set, a total of three biological replicates for each of the groups were used for proteomics analysis. Each biological sample comprised five individual plasma samples, which were equally mixed. High-abundance plasma proteins were depleted using the Human 14 Multiple Affinity Removal System Column (Agilent Technologies, Santa Clara, CA, United States) according to the manufacturer’s instructions. Low-abundance proteins were concentrated, freeze dried, and redissolved in 8 M UA buffer (8 M urea, 150 mM Tris HCl, pH 8.0). Protein concentrations of all three groups were determined through the Bradford assay. Subsequently, a total of 200 μg of samples from each group was lysed with 2 μg Lys-C at room temperature for 3 h. After dilution in a solution of 25 mM NH_4_HCO_3_ (Sigma, St. Louis, MO, United States) and 1.5 M urea, the protein mixture was digested by 2 μg sequencing-grade modified trypsin (Promega, Madison, WI, United States) at 37°C for 20 h.

### LC-MS/MS Analysis

The nano liquid HPLC system EASY-NLC1000 was applied for separation of the 5 μg tryptic peptide mixtures. In this system, mobile phase A was 0.1% formic acid in acetonitrile (2% acetonitrile), and mobile phase B was 0.1% formic acid in acetonitrile (84% acetonitrile). Chromatographic column Thermo EASY column SC200 150 μm ^∗^ 100 mm (RP-C_18_) was balanced by 100% mobile phase A solution. Approximately 5 μg of tryptic peptide mixture was loaded on to the column Thermo EASY column SC001 traps 150 μm ^∗^ 100 mm (RP-C_18_) (Thermo) and was separated by the chromatographic column at a flow rate of 400 nL/min using a linear gradient of B solution for 180 min. After separation, the tryptic peptide mixture was analyzed simultaneously with a Q-Exactive mass spectrometer (Thermo Finnigan) for 180 min. The MS was run in positive ion mode with a scan range of 300–1800 m/z. Each scan cycle consisted of one full MS scan in profile mode followed by 20 MS^2^ scans in centroid mode.

### Protein Identification and Relative Quantification

At first, nine original LC-MS/MS documents were split into three groups which were processed with Maxquant software (version number: 1.5.2.8) to get the label-free analysis. The spectra data of MS/MS were searched in the International Protein Index database (version 3.68, 91464 entries, Human). The following were the main parameters: 6 for the main search ppm, 2 for the missed cleavage, 20 for the MS/MS tolerance ppm, True set as de-isotopic, trypsin as digestion enzyme, ipi.human.3.68.fasta as database, carbamidomethyl as the fixed modification, Oxidation (M) and Acetyl (Protein N-term) as the variable modification, reverse as decoy database pattern, true set as LFQ, 2 for LFQ min ratio count, 2 min for match between runs, 0.01 for the peptide FDR, 0.01 for the protein FDR. A similarity search was performed in the UniProt database for *M.TB* with the following parameters: peptide mass tolerance for ±15 ppm and fragment mass tolerance for 20 mmu. Trypsin was used as the protein-cleaving enzyme, and the two missed cleavages were accepted. Carbamidomethylation of cysteine was designated as a fixed modification, and oxidation of methionine, acetylation on protein N-term were selected as variable modifications. The proteomics data were deposited in the iProx database^1^: IPX0001176000.

### Bioinformatics Analysis

Annotation of differentially expressed proteins including the CC, MF, and BP was obtained from the GO database^2^ and PANTHER analysis^3^. The biological signaling pathway analysis was performed with the KEGG database^4^. The reciprocity network of proteins identified was analyzed using the Search Tool for the Retrieval of Interacting Genes/Proteins (STRING) software^5^.

### Western Blot Analysis

Sodium dodecyl sulfate-polyacrylamide gels (12 and 8%) were prepared according to the molecular weight of target proteins. A total of 20 μg of samples was loaded onto these systems and transferred onto nitrocellulose filter membranes (Millipore, Corporation, Billerica, MA, United States) through electroblotting. The membranes were blocked with 3% BSA-TBST (10 mmol/L-Tris HCl, 150 mmol/L NaCl, 0.1% Tween 20 containing 5% skim milk) at room temperature for 30 min. Subsequently, membranes were incubated with the primary antibody including rabbit anti-CDH1 antibody (diluted 1:20000, Abcam, Cambridge, MA, United States), rabbit anti-CFH antibody (diluted 1:10000, Abcam), rabbit anti-alpha 1 antichymotrypsin antibody (diluted 1:5000, Abcam), rabbit anti-APOCIII antibody (diluted 1:1000, Abcam), rabbit anti-RBP4 antibody (diluted 1:5000, Abcam), rabbit anti-TF antibody (diluted 1:20000, Abcam), mouse anti-alpha 1 acid glycoprotein antibody (diluted 1:2000, Santa Cruz Biotechnology, Santa Cruz, CA, United States) and rabbit anti-CP antibody (diluted 1:10000, Abcam) at 4°C overnight. After washing five times membranes were incubated with horseradish peroxidase-conjugated goat anti-mouse IgG (diluted 1:5000, Santa Cruz Biotechnology), goat anti-rabbit IgG (Fc) (diluted 1:5000, Santa Cruz Biotechnology) and mouse anti-rabbit IgG (L) (diluted 1:5000, Santa Cruz Biotechnology) at room temperature for 40 min. Then they were reacted with enhanced chemiluminescence (ECL) solution (Millipore, Corporation, Billerica, MA, United States) according to the electrochemiluminescence kit instructions. Finally, the gray value of each band was measured by the TotalLab Quant software (TotalLab, Newcastle, United Kingdom).

### ELISA Analysis

The Human CDH1 ELISA kit (R&D Systems, Inc.; Minneapolis, MN, United States; the detection limit was 0.09 ng/mL), alpha 1 antichymotrypsin Human ELISA kit (Abcam, the detection limit was 1.722 ng/mL), APOCIII Human ELISA kit (Abcam, the detection limit was 0.001 μg/mL), Alpha 1 acid Glycoprotein Human ELISA kit (Abcam, the detection limit was 10.1 μg/mL), TF Human SimpleStep ELISA™ kit (Abcam, the detection limit was 292 pg/mL), Retinol Binding Protein 4 Human SimpleStep ELISA™ kit (Abcam, the detection limit was 19 pg/mL), Factor H Human ELISA Kit (Abcam, the detection limit was 0.2 ng/mL), and CP Human ELISA Kit (Abcam, the detection limit was 0.6 μg/mL) were used according to the manufacturers’ instructions to measure the concentrations of plasma proteins in the different groups.

### Statistical Analysis

Parametric data were expressed as the mean ± SD, and non-parametric data were expressed as the median (range). Continuous variables were tested using Student’s *t*-test or Mann–Whitney *U*-test, as appropriate. Categorized variables were analyzed using the Fisher’s exact test or Pearson’s chi-squared test. *P* < 0.05 was considered statistically significant. ROC curves were constructed to obtain the area under the curve (AUC) and evaluate the diagnostic values of each single biomarker. Logistic regression analysis was used to further evaluate the diagnostic accuracy of the combined plasma biomarkers. All data were analyzed using SPSS 17.0 (SPSS Inc., Chicago, IL, United States). A tree-structured data analytic technique referred to as CART analysis was used in the training set to classify the PTB group and non-TB groups (LTBI and HC groups). The CART software used a Gini splitting algorithm with 10-fold cross-validation that favored even splits and would not allow splits of nodes with five or fewer observations. The best decision tree was chosen using this algorithm, as previous study (Lu et al., 2011).

## Results

### Characteristics of the Study Population

A total of 398 participants were enrolled in this study, including 128 PTB patients, 125 LTBI individuals, 112 HCs, and 33 LC patients. The LC cases were consisted of adenocarcinoma (*n* = 20), squamous cell carcinoma (*n* = 8) and small cell LC (*n* = 5). The demographic characteristics of the study population are shown in **Table 1**.

**Table 1:** Demographic characteristics of the study population.

Study complex	Variables	PTB	LTBI	HC	LC	*P*-value^∗^	*P*-value^†^	*P*-value^‡^
Discovery set	*n*	15	15	15	–			
	Male/female	5/10	5/10	5/10	–	1.000	1.000	–
	Age (median, range)	28 (19-49)	31 (22-53)	29 (20-57)	–	0.523	0.806	–
	BMI (mean ± SD)	19.6 ± 2.0	20.9 ± 1.5	20.9 ± 1.7	–	0.058	0.077	–
	Smokers/non-smokers	0/15	1/14	1/14	–	1.000	1.000	–
	BCG vaccination, *n* (%)	13 (86.7)	15 (100%)	13 (86.7)	–	0.483	0.651	–
Training set	*n*	85	84	71	–			
	Male/female	55/30	45/39	40/31	–	0.141	0.286	–
	Age (median, range)	33 (16–65)	36 (20–65)	33 (19–60)	–	0.237	0.983	–
	BMI (mean ± SD)	20.3 ± 4.2	21.2 ± 3.9	20.8 ± 3.6	–	0.186	0.459	–
	Smokers/non-smokers	44/41	40/44	31/40	–	0.590	0.313	–
	BCG vaccination, *n* (%)	70 (82.3)	73 (86.9)	61 (85.9)	–	0.412	0.546	–
Blind testing set	*n*	28	26	26	33			
	Male/female	17/11	18/8	13/13	20/13	0.512	0.428	0.993
	Age (median, range)	25.5 (16–62)	29.5 (20–51)	26 (22–40)	63 (38–80)	0.966	0.256	<0.001
	BMI (mean ± SD)	19.8 ± 2.8	19.7 ± 2.6	20.9 ± 3.2	23.8 ± 2.8	0.919	0.173	<0.001
	Smokers/non-smokers	11/17	13/13	11/15	15/18	0.428	0.821	0.627
	BCG vaccination, *n* (%)	24 (85.7)	22 (84.6)	23 (88.5)	29 (87.9)	0.494	0.249	0.803

*n, number of subjects; SD, standard deviation; BMI, body mass index; PTB, pulmonary TB; LTBI, latent tuberculosis infection; HC, healthy control; LC, lung cancer. ^∗^Comparison between PTB and LTBI group; ^†^comparison between PTB and HC group; ^‡^comparison between PTB and LC group*.

In the discovery set, there were 15 PTB patients, and 15 age- and gender-matched LTBI individuals and HCs, respectively. An additional 85 PTB patients, 84 LTBI individuals, and 71 HCs were included in the training set for candidate biomarker validation and diagnostic model construction, while the remaining 28 PTB patients, 26 LTBI individuals, 26 HCs, and 33 LC patients were included in the blind testing set for diagnostic model validation (Supplementary Figure S1). There were no significant differences in the basic information, such as age, gender, and body mass index (BMI), between the PTB group and the control groups, except for the age and BMI between the PTB group and LC group in the blind testing set (*P* < 0.001) (**Table 1**).

### Label-Free Quantitative Proteomics Analysis

**FIGURE 1:**
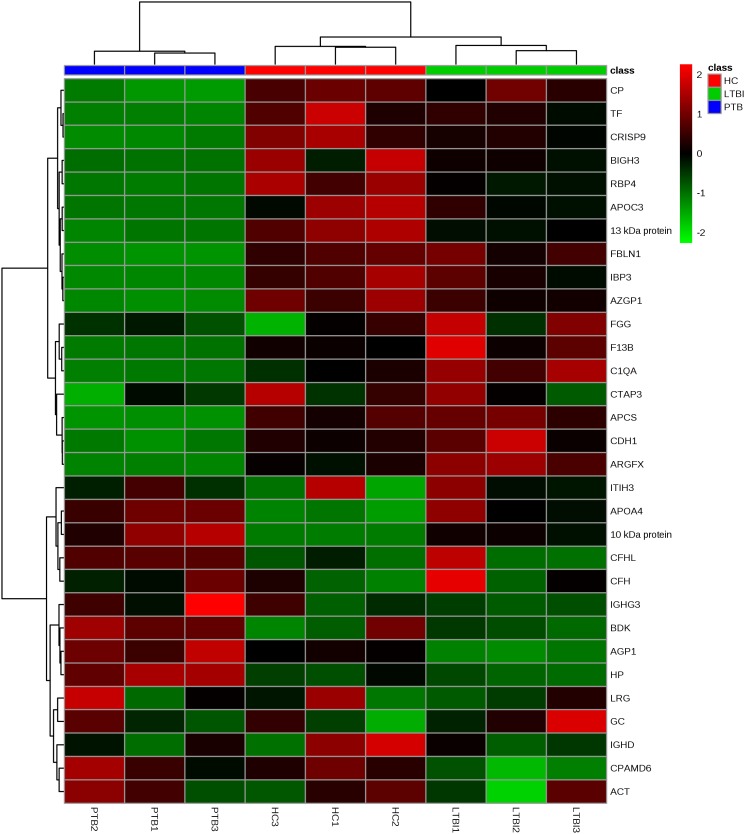
Hierarchical cluster analysis of the 31 differentially expressed proteins. Hierarchical cluster analysis was performed to determine whether the 31-protein profile could reflect the different statuses of *M.TB* infection. It showed that the nine pooled samples were successfully clustered into three groups, and each group matched exactly to the clinical grouping of PTB, LTBI, and HC. PTB, pulmonary TB; LTBI, latent tuberculosis infection; HC, healthy control.

Label-free quantitative proteomics was used to compare samples from the three groups (PTB, LTBI, and HC). In total, 229 non-redundant proteins were quantified based on the identification of one or more unique peptides. Differentially expressed proteins were defined as those that showed a fold change greater than 2.0 or less than 0.5 in relative abundance and a *P*-value < 0.05. Based on these criteria, there were 59 differentially expressed proteins between the PTB group and LTBI group, and 56 differentially expressed proteins between the PTB group and HC group, respectively. In the comparison between the PTB group and LTBI group, 26 proteins were up-regulated (>2-fold) and 33 proteins were down-regulated (<0.5-fold) in the PTB group. There were 26 up-regulated proteins (>2-fold) and 30 down-regulated proteins (<0.5-fold) in the PTB group when comparing with HC group. Comparison of PTB with the other two groups (PTB vs. LTBI and PTB vs. HCs) showed that a total of 31 overlapping proteins were significantly differentially expressed in plasma of PTB patients, and they presented the same trends when compare with LTBI and HC (**Table 2**). The intensity changes of the 31 differentially expressed proteins are shown as a heat map in **Figure 1**.

**Table 2:** Proteins identified by LC-MS/MS of PTB patients different from LTBI and HC individuals.

IPI number	Protein name	Gene	Uniprot	Mass/Da	PTB/LTBI		PTB/HC	

					Ratio	*P*-value	Ratio	*P*-value
IPI00744889	E-cadherin	CDH1	Q9UII7	99,694	0.2383	0.0487	0.3371	0.0198
IPI00007240	Coagulation factor XIII B chain	F13B	P05160	75,511	0.2004	0.0176	0.3325	0.0012
IPI00011264	Complement factor H-related protein 1	CFHL	Q03591	37,651	6.0731	0.0044	11.9184	0.0001
IPI00017601	Ceruloplasmin	CP	P00450	122,205	2.3079	0.0003	2.1999	0.0001
IPI00018219	Transforming growth factor-beta-induced protein ig-h3	BIGH3	Q15582	74,681	0.2084	0.0008	0.1137	0.0429
IPI00556155	Insulin-like growth factor binding protein 3 isoform a precursor	IBP3	P17936	31,674	0.362	0.0049	0.2691	0.0026
IPI00657670	Apolipoprotein C-III variant 1	APOC3	B0YIW2	12,816	0.084	0.0011	0.0468	0.0164
IPI00877703	Putative uncharacterized protein	FGG	C9JC84	52,338	2.0204	0.0123	2.9498	0.0054
IPI00022391	Serum amyloid P-component	APCS	P02743	25,387	0.2894	0.0021	0.3284	0.0018
IPI00022392	Complement C1q subcomponent subunit A	C1QA	P02745	26,017	0.1395	0.0019	0.2866	0.0108
IPI00022417	Leucine-rich alpha-2-glycoprotein	LRG	P02750	38,178	3.6325	0.0213	3.214	0.0279
IPI00022420	Retinol-binding protein 4	RBP4	P02753	23,010	0.3588	0.0004	0.1639	0.0035
IPI00884926	Alpha-1-acid glycoprotein 1	AGP1	B7ZKQ5	23,512	12.3226	0.0007	4.5748	0.0013
IPI00022445	Platelet basic protein	CTAP3/PPBP	P02775	13,894	2.46	0.0224	2.2171	0.0339
IPI00022463	Transferrin	TF	P02787	77,064	0.131	0.0009	0.0856	0.0133
IPI00025426	Isoform 1 of Pregnancy zone protein	CPAMD6	P20742	163,863	6.6994	0.0027	3.1198	0.0062
IPI00028413	Isoform 1 of Inter-alpha-trypsin inhibitor heavy chain H3	ITIH3	Q06033	99,849	2.8921	0.0030	3.2223	0.0044
IPI00029739	Isoform 1 of complement factor H	CFH	P08603	139,096	2.971	0.0010	3.5319	0.0007
IPI00829636	FLJ00382 protein (Fragment)	IGHD	P01880	42,353	3.1564	0.0017	2.2785	0.0111
IPI00166729	alpha-2-glycoprotein 1, zinc precursor	AZGP1	P25311	34,259	0.3167	0.00002	0.2225	0.0014
IPI00940451	59 kDa protein	IGHG3	P01860	41,287	6.525	0.0188	4.8085	0.0254
IPI00215894	Isoform LMW of kininogen-1	BDK	P01042	71,957	11.7329	0.0001	7.433	0.0013
IPI00889740	Fibulin 1	FBLN1	P23142	77,214	0.3419	0.0012	0.3271	0.0001
IPI00301143	Isoform 1 of Peptidase inhibitor 16	CRISP9/PI16	Q6UXB8	49,471	0.3792	0.0058	0.241	0.0076
IPI00304273	Apolipoprotein A-IV	APOA4	P06727	45,399	3.5902	0.0009	8.6196	0.0003
IPI00385762	Arginine-fifty homeobox	ARGFX	A6NJG6	35,617	0.2138	0.0015	0.3748	0.0022
IPI00550991	Alpha-1-antichymotrypsin	ACT/SERPINA3	P01011	47,651	3.7169	0.0049	3.2121	0.0047
IPI00555812	Gc-globulin	GC	P02774	52,964	2.3735	0.0303	3.5584	0.0127
IPI00641737	Haptoglobin	HP	P00738	45,205	13.781	0.0008	8.4063	0.0011
IPI00792393	10 kDa protein	–	–	101,160	5.7704	0.0123	192.1467	0.0064
IPI00796830	13 kDa protein	–	–	129,930	0.4777	0.0122	0.224	0.0043

*PTB, pulmonary TB; LTBI, latent tuberculosis infection; HC, healthy control*.

To investigate whether *M.TB* proteins were secreted in human plasma, the spectra data of MS/MS in our study were also searched in the UniProt database for *M.TB*. We identified 6, 6, and 2 *M.TB* proteins in active TB, LTBI, and HC samples, respectively. The six proteins identified in active TB samples were prpC, pbpB, rpsN, pyrR, mntH, and Rv0102, while the six proteins identified in LTBI samples were relA, vapB18, clpP2, kasA, Rv0064, and Rv2633c. Two proteins, including aceAa and dxr, were detected in HC samples.

### Bioinformatics Analysis of Differentially Expressed Proteins

**FIGURE 2:**
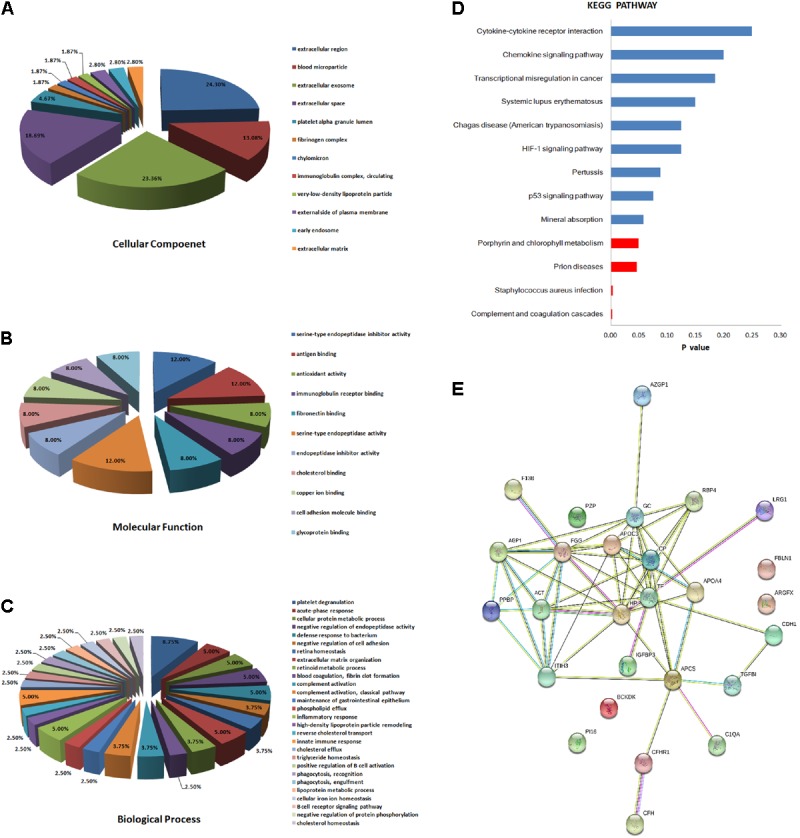
Bioinformatics analysis of the differentially expressed proteins. **(A)** Cellular component (GO analysis); **(B)** molecular function (GO analysis); **(C)** biological process (GO analysis); **(D)** KEGG pathway analysis; **(E)** function network analysis of differentially expressed proteins using STRING software.

We classified the 31 differentially expressed proteins by GO analysis as CC, BP, and MF. The results showed that the majority of the proteins have an extracellular distribution, involving extracellular region (24.30%), extracellular exosome (23.36%), and extracellular space (18.69%) (**Figure 2A**). According to the analysis of MF, the differentially expressed proteins were categorized into different groups. Expectedly, the top category detected was related to binding, including antigen binding (12.00%), fibronectin binding (8.00%), cholesterol binding (8.00%), copper ion binding (8.00%), cell adhesion molecular binding (8.00%) and glycoprotein binding (8.00%). The other proteins were categorized into antioxidant activity (8.00%), serine-type endopeptidase activity (12.00%) and serine-type endopeptidase inhibitory activity (12.00%) (**Figure 2B**). In addition, the important proteins identified in the PTB group that were significantly up or down-regulated were categorized into a diverse set of functional groups, including acute-phase response (5.00%), defense response to bacterium (5.00%), inflammatory response (5.00%) and innate immune response (5.00%), and so on (**Figure 2C**).

Kyoto Encyclopedia of Genes and Genomes enrichment analysis was then implemented to test proteomics pathway enrichment. KEGG enrichment indicated that complement and coagulation cascades, *Staphylococcus aureus* infection, prion diseases, and porphyrin and chlorophyll metabolism are significantly associated with active PTB (**Figure 2D**). The protein–protein functional network diagram analysis also demonstrated that most of these differentially expressed proteins closely interacted with each other (**Figure 2E**).

### Validation of Identified Proteins by Western Blot

**FIGURE 3:**
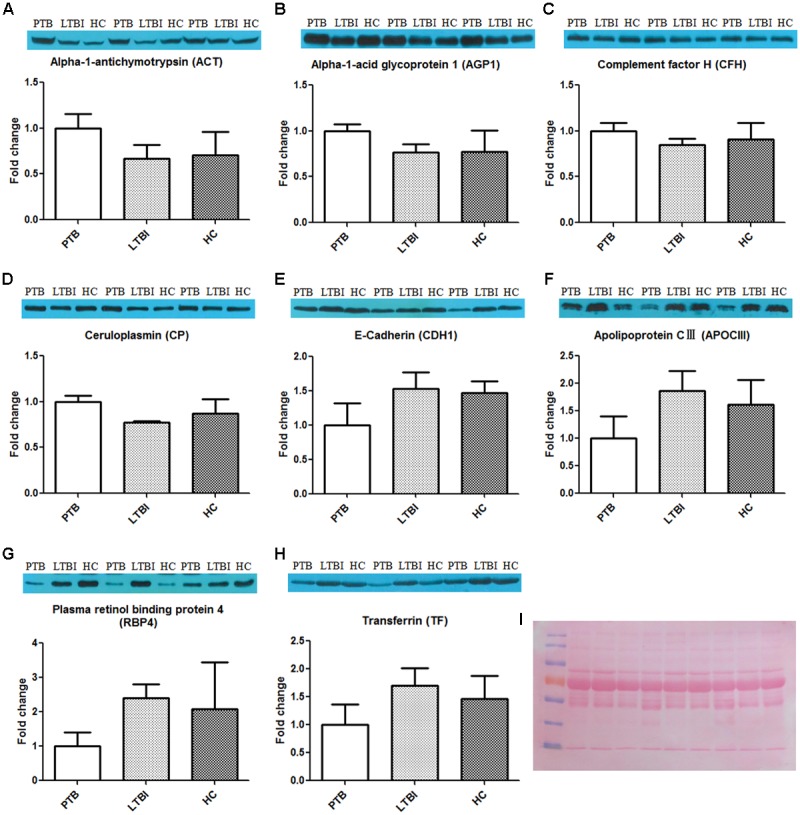
Validation of the differentially expressed proteins by western blot. The eight differential proteins were selected to be validated by western blot, using the nine pooled samples in the discovery set. All these proteins showed the same expression pattern with proteomics analysis. ACT **(A)**, AGP1 **(B)**, CFH **(C)**, and CP **(D)** were up-regulated in the PTB group when compared with the LTBI and HC groups. CDH1 **(E)**, APOCIII **(F)**, RBP4 **(G)**, and TF **(H)** were down-regulated in the PTB group when compared with the LTBI and HC groups. **(I)** The nitrocellulose filter membranes were stained with Ponceau S to ensure equal loading between each sample in western blot analysis. PTB, pulmonary TB; LTBI, latent tuberculosis infection; HC, healthy control.

After synthesizing the results, including fold change and bioinformatics data, as well as considering the biological functions of these differentially expressed proteins associated with infection and immunity, 4 up-regulated proteins (ACT, AGP1, CFH, and CP) and 4 down-regulated proteins (CDH1, APOCIII, RBP4, and TF) were selected to be validated by western blotting, using the pooled samples in the discovery set (**Figure 3**). During western blotting analysis, the nitrocellulose filter membranes were stained with Ponceau S to ensure equal loading between each sample (Wang et al., 2012). Based on the western blotting results, all eight proteins exhibited the same expression pattern as indicated in the results of proteomics analysis. ACT, AGP1, CFH, and CP were up-regulated in PTB patients compared with the other two groups. The average PTB/LTBI ratios of these proteins were 1.50, 1.30, 1.18, and 1.29, respectively. The PTB/HC ratios were 1.41, 1.29, 1.10, and 1.15, respectively. CDH1, APOCIII, RBP4, and TF were down-regulated in PTB patients. The average PTB/LTBI ratios were 0.65, 0.53, 0.42, and 0.59, respectively. The PTB/HC ratios were 0.68, 0.62, 0.48, and 0.68, respectively.

### Validation of Differentially Expressed Proteins in Training Set by ELISA

**FIGURE 4:**
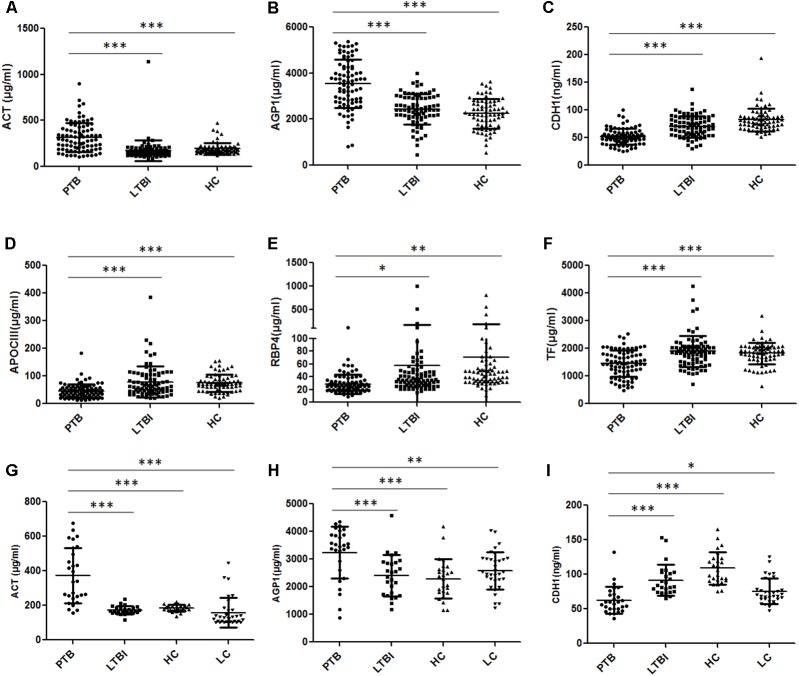
Validation of the differentially expressed proteins by ELISA. **(A–F)** Validation of the six differentially expressed proteins in the training set. Significant differences in ACT, AGP1, CDH1, APOCIII, RBP4, and TF were noted in the comparison of the PTB group with the LTBI and HC group, and the expression trends of these proteins were consistent with the proteomics results. **(G–I)** Validation of the three differentially expressed proteins in the blind test set. Significant differences in ACT, AGP1, and CDH1 were noted in the comparison of the PTB group with non-TB groups (LTBI, HC, and LC group). Data presented as mean ± *SD*. ^∗^*P* < 0.05, ^∗∗^*P* < 0.01, ^∗∗∗^*P* < 0.001. PTB, pulmonary TB; LTBI, latent tuberculosis infection; HC, healthy control; LC, lung cancer.

A total of 240 individuals (85 PTB patients, 84 LTBI individuals, and 71 HCs) were recruited in this training set. In this phase, 80 samples (27 PTB patients, 30 LTBI individuals, and 23 HCs) were randomly selected to initially verify the eight proteins by ELISA. CFH and CP results were inconsistent with the proteomics results and were excluded from subsequent validation. The other six proteins were further confirmed in the remaining 160 samples (58 PTB patients, 54 LTBI individuals, and 48 HCs). Statistically significant differences in plasma ACT, AGP1, APOCIII, CDH1, RBP4, and TF were noted in the PTB group, when compared with the LTBI group and HC group (**Figures 4A–F** and Supplementary Table S1). Furthermore, the trends of the plasma expression levels of these proteins based on ELISA validation were coincident with the proteomics results. The plasma concentrations of ACT and AGP1 were significantly increased in the PTB group samples compared with those in the LTBI group samples (*P* < 0.001 and *P* < 0.001, respectively) and HC group samples (*P* < 0.001 and *P* < 0.001, respectively), while the plasma concentrations of CDH1, APOCIII, RBP4, and TF were significantly decreased in the PTB group samples than that in the LTBI group samples (*P* < 0.001, *P* < 0.001, *P* < 0.05, and *P* < 0.001, respectively) and HC group samples (*P* < 0.001, *P* < 0.001, *P* < 0.01, and *P* < 0.001, respectively).

### ROC Analysis

Receiver operating characteristic (ROC) analysis was performed to evaluate the sensitivity and specificity of the six proteins in the training set (Supplementary Figure S2 and **Table 3**). The AUC values of ACT, AGP1, CDH1, APOCIII, RBP4, and TF were 0.835, 0.816, 0.784, 0.721, 0.724, and 0.723, respectively, when discriminating between the PTB group and LTBI group. The plasma protein ACT had the best ability to distinguish between PTB and LTBI, followed by the proteins AGP1 and CDH1. The AUC values of ACT, AGP1, CDH1, APOCIII, RBP4, and TF were 0.762, 0.856, 0.925, 0.793, 0.823, and 0.723, respectively, when discriminating between the PTB group and HC group. CDH1 was the best protein for distinguishing the PTB group from the HCs group. Logistic regression with forward stepwise analysis indicated that APOCIII and RBP4 were excluded, and CDH1, TF, ACT, and AGP1 were included in the diagnostic model for discrimination of the PTB group from the LTBI group. The AUC value of this diagnostic model reached as high as 0.946, and the model exhibited 82.3% sensitivity and 92.8% specificity in discriminating the PTB group from the LTBI group. In addition, logistic regression with forward stepwise analysis indicated that TF and RBP4 were excluded, and APOCIII, CDH1, ACT, and AGP1 were included in the diagnostic model for discrimination of the PTB group from the HC group. The AUC value of this diagnostic model reached 0.989, and the model exhibited 96.5% sensitivity and 95.8% specificity in discriminating the PTB group from the HC group. These results revealed that the combination of the proteins enables higher diagnostic capacity than each single protein.

**Table 3:** The AUC, sensitivity and specificity of the six differentially expressed proteins and the panels in discriminating PTB patients from LTBI individuals, and from HCs, using logistic regression analysis.

Category	Parameters	ACT	AGP1	CDH1	APOCIII	RBP4	TF	Panel^∗^
PTB vs. LTBI	Sensitivity (%)	68.2	63.5	65.9	85.9	68.2	72.3	82.3
	Specificity (%)	92.9	91.8	78.6	47.6	69.1	61.9	92.8
	AUC	0.835	0.816	0.784	0.721	0.724	0.723	0.946
PTB vs. HC	Sensitivity (%)	69.4	63.5	80.0	77.6	65.9	62.3	96.5
	Specificity (%)	88.7	94.4	92.9	73.2	92.9	81.7	95.8
	AUC	0.762	0.856	0.925	0.793	0.823	0.723	0.989

*PTB, Pulmonary tuberculosis; LTBI, latent tuberculosis infection; HC, healthy control. AUC, area under the ROC curve. ^∗^Panel: a combination of the plasma CDH1, TF, ACT, and AGP1 in discriminating the PTB group from the LTBI group; a combination of the plasma APOCIII, CDH1, ACT, and AGP1 in discriminating the PTB group from the HC group*.

### A Simple Diagnostic Model for Distinguishing the PTB Group and Non-TB Group

**FIGURE 5:**
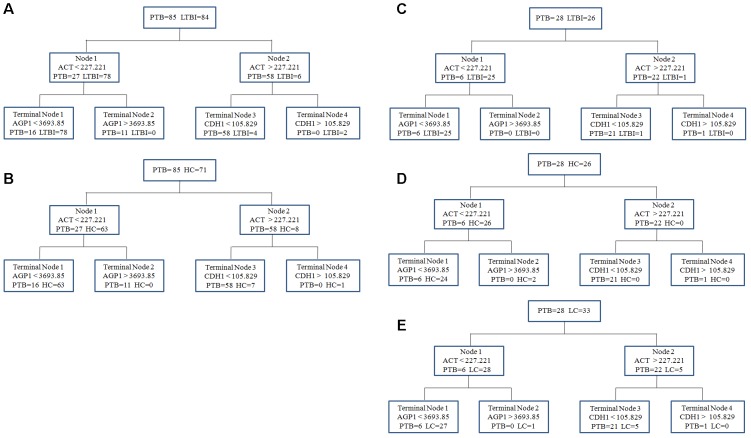
The diagnostic model for distinguishing PTB and non-TB individuals using the classification and regression tree (CART) analysis. **(A,B)** ACT, AGP1, and CDH1 were incorporated to establish a diagnostic model using CART analysis in the training set. The tree structure and the cutoff values of each node were consistent in discriminating the PTB group from the LTBI and HC groups. **(C–E)** Validation of the diagnostic model in the blinding test set. Similar with the training set, the diagnostic model performed well to discriminate the PTB group from the three control groups. PTB, pulmonary TB; LTBI, latent tuberculosis infection; HC, healthy control; LC, lung cancer.

To obtain a simple and visual diagnostic model and facilitate clinical application, we subjected these six proteins to CART analysis to determine the ideal protein combination and to optimize the discrimination between PTB and non-TB. This analysis demonstrated that a combination of ACT, AGP1, and CDH1 presented the best discriminating capacity (**Figures 5A,B**). When discriminating the PTB group and LTBI group, this classification tree yielded a sensitivity of 81.2% (69/85), a specificity of 95.2% (80/84), and an accuracy of 88.2% (149/169). In addition, this combination of ACT, AGP1, and CDH1 demonstrated good capacity for discrimination between the PTB group and HC group. The sensitivity, specificity, and accuracy of this combination was 81.2% (69/85), 90.1% (64/71), and 85.2% (133/156), respectively.

### Blind Test of the Diagnostic Model

In the independent blind test, we validated the diagnostic accuracy of the ACT-AGP1-CDH1 combination with identification of PTB patients (*n* = 28) and non-TB individuals, including LTBI individuals (*n* = 26), HCs (*n* = 26) and LC patients (*n* = 33). The expression of three proteins of interest was also examined by ELISA. Statistically significant differences of these three proteins were noted in the PTB groups when compared with that in the LTBI, HC and LC groups (**Figures 4G–I** and Supplementary Table S2). Similar to the training set, the diagnostic model effectively discriminated the PTB group and the other three groups (**Figures 5C–E**). According to the optimal cutoff value yielded in the diagnostic model, it could classify 85.2% individuals (46/54) with a sensitivity of 75.0% (21/28) and a specificity of 96.1% (25/26) in discriminating the PTB patients from the LTBI individuals. Moreover, it presented a sensitivity of 75.0% (21/28), a specificity of 92.3% (24/26), and an accuracy of 83.3% (45/54) when discriminating the PTB group from the HCs group. Furthermore, it showed a sensitivity of 75.0% (21/28), a specificity of 81.8% (27/33), and an accuracy of 78.7% (48/61) in discriminating the PTB group from the LC group.

## Discussion

The human immune response to *M.TB* infection is highly complicated and multifaceted. Identifying the immunologic characteristics of different infection statuses (LTBI or active TB) will facilitate early diagnosis of active TB or LTBI and the understanding of the pathogenesis from latent infection to TB reactivation. In this study, a label-free quantitative proteomics technique was applied to compare the proteomic profiles of the PTB, LTBI, and HC individuals, and we detected distinct plasma protein biomarkers of PTB, LTBI, and HCs. A total of 31 differentially expressed proteins were identified in the PTB patients, compared with LTBI and HCs. Most importantly, plasma ACT, AGP1, and CDH1 were combined to construct a simple and visual diagnostic model, which had relatively high diagnostic accuracy for distinguishing the active TB patients from non-TB groups.

Label-free quantitative proteomics has obviated the requirement for protein staining or peptide labeling, and it is a powerful technique with a high capacity for multiplexing (simultaneously measuring multiple biomarkers), a modest peranalyte sample volume, very low technical variability, and higher dynamic range and proteome coverage capacity (Sandin et al., 2015), in comparison with other proteomic technologies. This technology has been widely used in identifying potential biomarkers of cancers (Fan et al., 2015; Gamez-Pozo et al., 2015), neurodegenerative diseases (Perrin et al., 2013), renal transplantation (Welberry Smith et al., 2013), and endocrine system and metabolic diseases (Moulder et al., 2015). The available information on proteomics analysis between active TB and LTBI was limited until now. Most of the previous studies on TB biomarker screening were focused on the differential analysis between active TB and the other pulmonary diseases (COPD, LC, or pneumonia), or between active TB and HC (Liu et al., 2011, 2013, 2014; Song et al., 2014; Xu et al., 2014, 2015). Only one study has characterized the plasma proteins in children at different *M.TB* infection stage (active TB and LTBI), and four proteins (XRCC4, PCF11, SEMA4A, and ATP11A) were detected and confirmed between active TB and LTBI using proteomics analysis and followed western blot analysis (Li et al., 2017). However, these four proteins were not detected in our study, possibly because of the considerable difference in immune response between adults and children.

A total of 31 differentially expressed proteins were identified in both PTB vs. LTBI and PTB vs. HC comparisons. These proteins could be closely related to the status of active TB. GO analysis clarified the biological significance of these 31 differentially expressed proteins. CC analysis by GO revealed that most of the proteins were located in the extracellular region, indicating that they are the secretory proteins. The secretory proteins play important roles in mediating intracellular signal transduction in host defense and inflammation development in TB. Interestingly, some extracellular exosome proteins (23.36%) were detected in the analysis. Since we did not deplete any organelles or subcellular structures in plasma before LC-MS/MS analysis, it is reasonable that exosome proteins were detected in our proteomics analysis. MF analysis indicated that proteins with binding activity accounted for a large proportion, consistent with a previous study that screened the differentially expressed serum proteins in active TB compared with HC (Xu et al., 2014). BP analysis revealed that proteins involved in the human immune response process accounted for a large proportion. *M.TB* infection and further active TB development are accompanied with the interaction between host immune response and bacterial invasion; thus, the expression of proteins participating in the immune response must be significantly different between *M.TB* infection and non-infection. This explains why proteins involved in the human immune response process were detected at a larger proportion in active TB status.

Furthermore, *M.TB* secreted proteins were detected in our study, which may be derived from the plasma exosomes. Exosomes represent a promising research tool for TB diagnosis and treatment because they are released from various cells containing valuable biochemical information (proteins, lipids, and nucleic acids) relating to disease. Recently, many reports have suggested *M.TB* secreted proteins or nucleic acid in plasma/serum exosomes as candidate diagnostic markers for TB (Giri et al., 2010; Kruh-Garcia et al., 2014; Lee et al., 2015; Mehaffy et al., 2017). The spectra data of MS/MS in our study were also searched for *M.TB* protein, and a total of 6, 6, and 2 *M.TB* proteins were identified in PTB, LTBI, and HC samples, respectively. Since our study was focused on the screening of human plasma proteins for discriminating PTB from LTBI, and we did not isolate the plasma exosomes, the proteome was predominately comprised of host proteins with only a small portion of the collected spectra data corresponding to mycobacterial peptides. Further research is needed to systematically explore the *M.TB* secreted proteins in plasma/serum exosomes and further elucidate the role of these *M.TB* proteins in the pathogenesis from LTBI to active TB.

The six plasma proteins, ACT, AGP1, CDH1, APOCIII, RBP4, and TF, showed significantly different expression levels in PTB patients compared with those in LTBI and HC individuals. Specifically, ACT and AGP1 concentrations were significantly increased in the plasma of patients with PTB compared with those of the other two groups. These two proteins are acute-phase proteins involved in various inflammatory conditions. A previous study suggested that the serum ACT level was generally higher among active TB patients, which was in line with our results (Song et al., 2014). ACT acts as a regulatory enzyme that primarily inhibits neutrophil elastase activity and thus protects tissues from proteolytic damage after inflammation (Stoller and Aboussouan, 2012). The deficiency of ACT is associated with the development of pulmonary emphysema (Daniel et al., 1988). In the active TB, the elevated plasma ACT level might be a protective factor to prevent or defer lung damage. It has been reported that AGP1 was rapidly produced in the infected lung at the early *M.TB* infection stage, and 10-fold higher concentrations were detected during the progressive phase of *M.TB* infection (Martinez Cordero et al., 2008). Furthermore, the distinct expression pattern of the serum AGP1 in TB patients was useful in the differential diagnosis of bacterial lung infections (Fassbender et al., 1995). Similar to our results, it has been reported that AGP1 levels were elevated in active TB patients and may be a potential marker for low response to anti-TB treatment (Hernandez-Pando et al., 1998). A previous study also determined that LTBI individuals with an elevated AGP level were essentially at the early phase of active TB (Jensen et al., 2013). CDH1 belongs to the family of adhesion molecules known as ‘cadherins,’ which mediate the interaction and adhesion between epithelial cells and maintain the integrity of the organization (Takeichi, 1991). It has been reported that CDH1 is a potential epithelial master gene, and the decreased level of CDH1 is associated with the onset of EMT (Kim et al., 2014). A recent study indicated that the EMT of mesothelial cells occurred in TB pleurisy, together with a reduction in the CDH1 level (Kim et al., 2011). Furthermore, the down-regulated expression of CDH1 was detected in *M.TB* (*H37Rv*)-infected THP-1 cells and further induced the EMT process (Gupta et al., 2015). All these results were consistent with our results showing that the plasma CDH1 level was reduced in PTB patients. APOCIII is a very low-density lipoprotein, and one of the lipid carriers associated with lipid metabolism. It inhibits the activity of lipoprotein lipase and was positively associated with higher concentration of serum/plasma triglycerides in circulation (Bobik, 2008). A previous study has suggested that PTB patients have lower serum lipid levels than those of HC, which was correlated to the fact that PTB patients were generally losing weight (Deniz et al., 2007). This phenomenon might be related to the reduced APOCIII level; we indeed detected a reduced plasma APOCIII level in PTB patients in our study. Consistent with our results, Liu et al. (2014) also identified that APOCIII was down-regulated in Traditional Chinese Medicine (TCM) syndromes of TB. Furthermore, one of the three down-regulated ‘MS peak’ that exhibited high diagnostic accuracy was identified as an isoform of APOCIII in smear-positive PTB patients (Liu et al., 2015). It has been reported that RBP4 modulates pathophysiological processes during *M.TB* infection (Tanaka et al., 2011). Two related TB proteomics studies have shown that RBP4 was reduced in the whole-blood supernatants from patients with active TB and in the plasma of patients with PTB; Therefore, RBP4 was identified as a candidate biomarker for active TB (Tanaka et al., 2011; Xu et al., 2014). These studies directly confirmed our results that the plasma RBP4 was decreased in PTB patients, although the control groups in the two studies were not LTBI individuals. The function of TF is to transport iron, which is critical for *M.TB* growth (Boelaert et al., 2007). Iron homeostasis was associated with TB progression, and lower concentration of TF has been identified as a risk factor for progression to TB (Minchella et al., 2015). In this study, the lower plasma TF level might be a reason for the progression to active PTB, compared with that of the LTBI individuals and HC without any symptoms. Based on these analyses, we hypothesized that the six proteins may be candidate biomarkers for distinguishing PTB patients from LTBI and HCs. Furthermore, these proteins may be promising markers to explore the pathogenesis of TB and the transition from latent infection to active TB.

We evaluated the diagnostic capacity of the six proteins and found that all the proteins had high AUC values (>0.7). Logistic regression analysis showed that the panel of ACT_AGP1_CDH1_TF could improve the diagnostic accuracy to 87.6% for PTB vs. LTBI, and the panel of ACT_AGP1_CDH1_APOCIII could improve the diagnostic accuracy to 96.2% for PTB vs. HC. These panels of proteins exhibited excellent differential capacity for discriminating PTB from LTBI or HCs. However, one shortcoming of these panels was that the distinct pattern of proteins is required to distinguish active TB and the two control groups, which may cause difficulty for use in clinical settings. We cannot know whether an individual was a LTBI or healthy control prior to clinical examinations; therefore, we would not know the optimal panel to use to diagnose active TB. In order to obtain a simple visual model with relatively high accuracy for diagnosing active TB, we used the tree-structured data analytic technique CART to select the best biomarker panel and construct the model. Consequently, ACT, AGP1, and CDH1 were finally chosen to build the decision trees for distinguishing the PTB group and the two control groups. The diagnostic accuracies were more than 85% in both PTB vs. LTBI and PTB vs. HC differentiation, with high specificity for ruling out the possibility of active TB. Since active TB is a benign disease, and arbitrary anti-TB treatment will result in severe adverse reactions, a relatively higher-specificity diagnosis method will be preferred by clinicians and patients. Furthermore, the model performed well in a blind testing set, and it also presented a relatively good sensitivity and specificity in distinguishing PTB patients and LC patients. These results indicated that this diagnostic model may be promising for discriminating PTB from non-TB.

To our knowledge, this is the first study to identify candidate plasma biomarkers for distinguishing PTB from latent infection in adults using a label-free proteomics technique and further validated in two independent clinical settings with a relatively larger sample size. However, there are still some limitations in our study. First, there was a lack of controls with diverse pulmonary diseases other than TB in the discovery set, although LC patients were included in the blind testing set to validate the diagnostic model. Furthermore, considering the small number of samples in the discovery set, the pooled samples were used in the proteomics analysis in order to eliminate the effect of inter-individual difference. However, some differentially expressed proteins between TB cases and controls may have been obscured after pooling, which cannot be avoided. Larger numbers of independent samples in proteomics analysis may be the optimal method to avoid this dilemma. In addition, although the total number of samples in our study was sufficiently large, the sample size in the blind testing set for model validation was still moderate. Further studies with increased sample size will be needed to validate this model. Lastly, since a part of TB patients in clinical were smokers, about half of the enrolled participants in the following validation and blind test sets were smokers, which was discrepant from the discovery set, in which most participants were non-smokers. However, the control groups enrolled in the validation and blind test sets were matched in smoking status to avoid the potential interference of smoking. Furthermore, the diagnostic model presented an accuracy of 91.8 and 90.2% in discriminating the PTB group from the LTBI and HC groups in the validation set, respectively, and an accuracy of 86.7, 87.5, and 77.1% in discriminating the PTB group from the LTBI, HC, and LC groups, which indicated that the discrepancy of smoking status between the discovery set and the following validation and blind test sets may not have led to bias in the analysis.

## Conclusion

Our study uncovered plasma proteomic profiles of different *M.TB* infection statuses in adults, and identified 31 differentially expressed proteins between PTB patients and LTBI or HC individuals. Furthermore, a new diagnostic model consisting of ACT, AGP1, and CDH1 was established and presented a relatively good capacity in discriminating PTB patients from LTBI individuals. These results provide a new potential diagnostic signature for distinguishing PTB and latent infection, and may facilitate better understanding of the pathogenesis involved in the transition from latent infection to TB activation.

## Author Contributions

LP and ZoZ designed the experiments. HS, LP, HJ, QS, and RW conducted the experiments. ZhZ, MG, MH, JW, BD, and AX enrolled the subjects. HS and LP analyzed the data and wrote the paper.

## Conflict of Interest Statement

The authors declare that the research was conducted in the absence of any commercial or financial relationships that could be construed as a potential conflict of interest.

## Footnotes

1 http://www.iprox.org/

2 http://www.geneontology.org/

3 http://www.pantherdb.org/

4 https://www.genome.jp/kegg/pathway.html

5 http://string.embl.de/

## Abbreviations

ACT: alpha-1-antichymotrypsin
AGPI: alpha-1-acid glycoprotein 1
APOCIII: apolipoprotein CIII
AUC: area under the ROC curve
BP: biological process
CART: classification and regression tree
CC: cellular component
CDH1: E-cadherin
CFH: complement factor H
CP: ceruloplasmin
EMT: epithelial to mesenchymal transition
GO: gene ontology
HCs: healthy controls
KEGG: Kyoto Encyclopedia of Genes and Genomes
LC: lung cancer
LFQ: label-free quantification
LTBI: latent tuberculosis infection
*M.TB*: *Mycobacterium tuberculosis*
MF: molecular function
PTB: pulmonary TB
RBP4: plasma retinol binding protein 4
ROC: receiver–operator characteristic
SD: standard deviation
TB: tuberculosis
TF: transferrin
TST: tuberculin skin test.
