# Supplementary material for: Label-Free Quantitative Proteomics Identifies Novel Plasma Biomarkers for Distinguishing Pulmonary Tuberculosis and Latent Infection
Source: Front Microbiol. 2018 Jun 13;9:1267. doi: 10.3389/fmicb.2018.01267 (PMC6008387; doi:10.3389/fmicb.2018.01267)
Supplement: Supplementary file 1 [file Table_1.DOCX]

**Supplementary Table 1. The expression level of the 6 differentially expressed proteins in the training set.**

| Proteins | PTB group (n = 85) | LTBI group (n = 84) | HC group (n = 71) | *P*-value* | *P*-value ^†^ |
| --- | --- | --- | --- | --- | --- |
| ACT (μg/ml) | 318.2 ± 156.5 | 172.7 ± 114.4 | 194.8 ± 62.7 | < 0.001 | < 0.001 |
| AGP1 (μg/ml) | 3547.2 ± 1042.3 | 2413.1 ± 707.7 | 2244.4 ± 654.4 | < 0.001 | < 0.001 |
| CDH1 (ng/ml) | 51.8 ± 14.2 | 69.9 ± 19.1 | 82.4 ± 20.7 | < 0.001 | < 0.001 |
| APOCIII (μg/ml) | 45.7 ± 25.6 | 78.3 ± 55.9 | 74.3 ± 30.4 | < 0.001 | < 0.001 |
| RBP4 (μg/ml) | 28.6 ± 15.2 | 57.4 ± 117.6 | 70.4 ± 118.1 | 0.026 | 0.0015 |
| TF (μg/ml) | 1453.7 ± 481.4 | 1891.7 ± 559.5 | 1820.9 ± 389.9 | < 0.001 | < 0.001 |

* Comparison between PTB and LTBI group;

^†^ Comparison between PTB and HC group;

Data presented as mean ± SD

PTB, pulmonary TB; LTBI, latent tuberculosis infection; HC, healthy control.
