# Supplementary material for: Label-Free Quantitative Proteomics Identifies Novel Plasma Biomarkers for Distinguishing Pulmonary Tuberculosis and Latent Infection
Source: Front Microbiol. 2018 Jun 13;9:1267. doi: 10.3389/fmicb.2018.01267 (PMC6008387; doi:10.3389/fmicb.2018.01267)
Supplement: Supplementary file 2 [file Table_2.DOCX]

**Supplementary Table 2. The expression level of the 3 candidate biomarkers in the blind testing set.**

| Proteins | PTB group (n = 28) | LTBI group (n = 26) | HC group (n = 26) | LC group (n = 33) | *P*-value* | *P*-value ^†^ | *P*-value ^‡^ |
| --- | --- | --- | --- | --- | --- | --- | --- |
| ACT (μg/ml) | 371.5 ± 160.2 | 172.2 ± 24.2 | 183.8 ± 19.5 | 157.9 ± 85.4 | < 0.001 | < 0.001 | < 0.001 |
| AGP1 (μg/ml) | 3237.6 ± 939.6 | 2406.2 ± 744.7 | 2291.3 ± 717.8 | 2583.4 ± 682.5 | < 0.001 | < 0.001 | 0.003 |
| CDH1 (ng/ml) | 62.5 ± 19.6 | 91.2 ± 22.5 | 108.7 ± 23.5 | 75.3 ± 18.5 | < 0.001 | < 0.001 | 0.011 |

* Comparison between PTB and LTBI group;

^†^ Comparison between PTB and HC group;

^‡^ Comparison between PTB and LC group;

Data presented as mean ± *SD*

PTB, pulmonary TB; LTBI, latent tuberculosis infection; HC, healthy control; LC, lung cancer.
