# Supplementary figures and images for: Label-Free Quantitative Proteomics Identifies Novel Plasma Biomarkers for Distinguishing Pulmonary Tuberculosis and Latent Infection
Source: Front Microbiol. 2018 Jun 13;9:1267. doi: 10.3389/fmicb.2018.01267 (PMC6008387; doi:10.3389/fmicb.2018.01267)

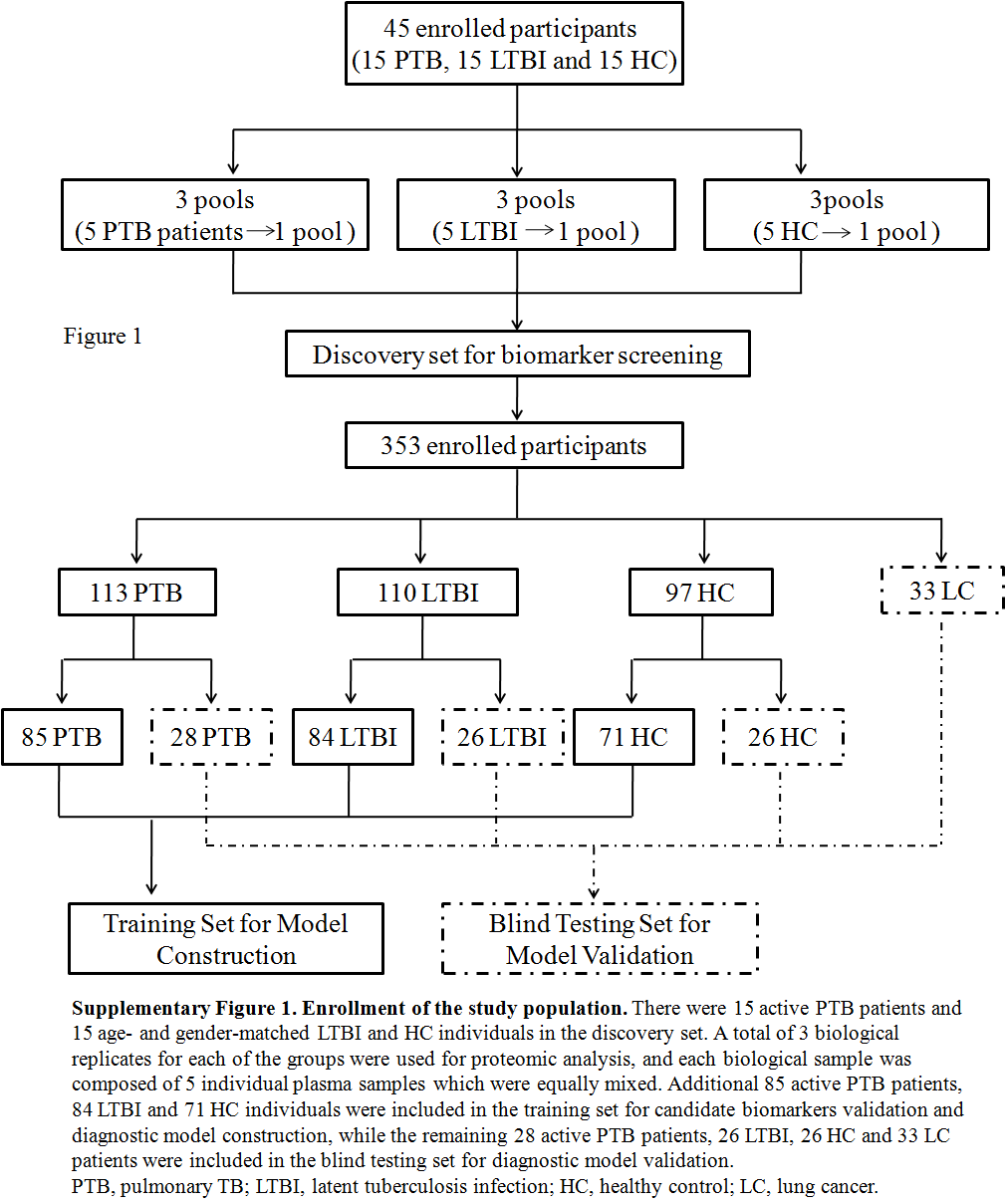

Supplement: Supplementary file 3 [file Image_1.TIF]

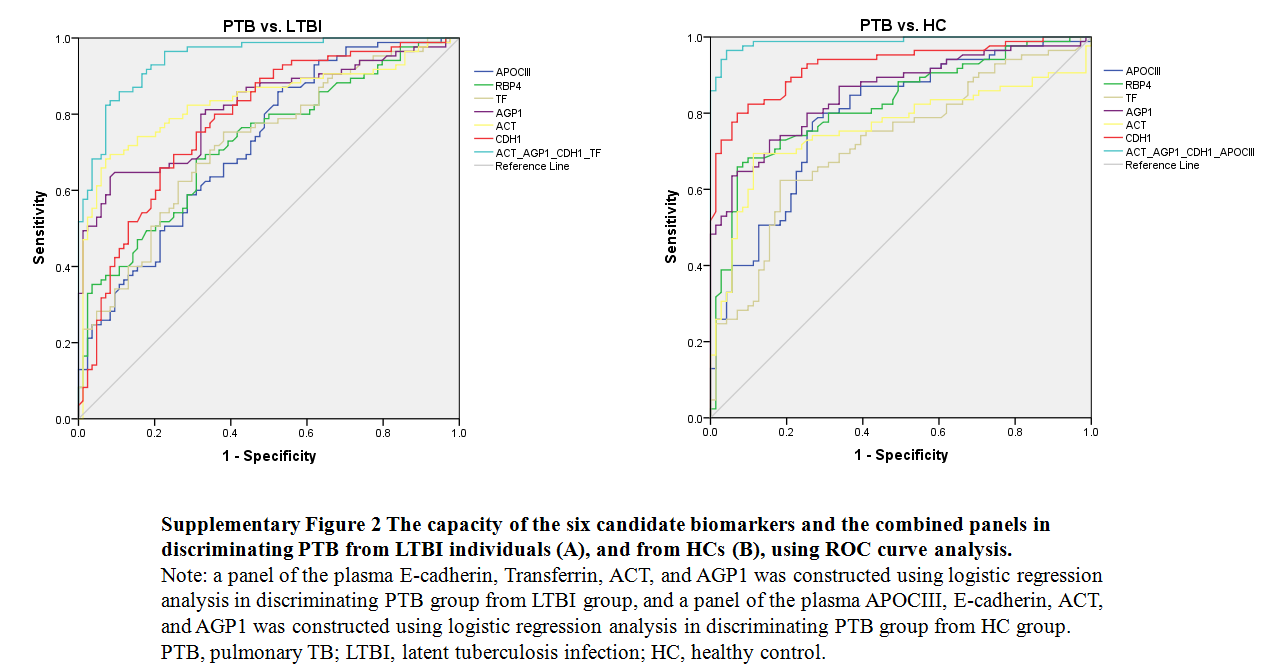

Supplement: Supplementary file 4 [file Image_2.TIF]
